# Supplementary material for: Single molecule studies of the native hair cell mechanosensory transduction complex
Source: bioRxiv. 2023 Dec 12:2023.12.11.571162. Preprint. [Version 1] doi: 10.1101/2023.12.11.571162 (PMC10760052; doi:10.1101/2023.12.11.571162)
Supplement: 1 [file NIHPP2023.12.11.571162V1-supplement-1.pdf]

bioRxiv preprint doi: https://doi.org/10.1101/2023.12.11.571162; this version posted December 12, 2023. The copyright holder for this preprint (which was not certified by peer review) is the author/funder, who has granted bioRxiv a license to display the preprint in perpetuity. It is made available under aCC-BY 4.0 International license.

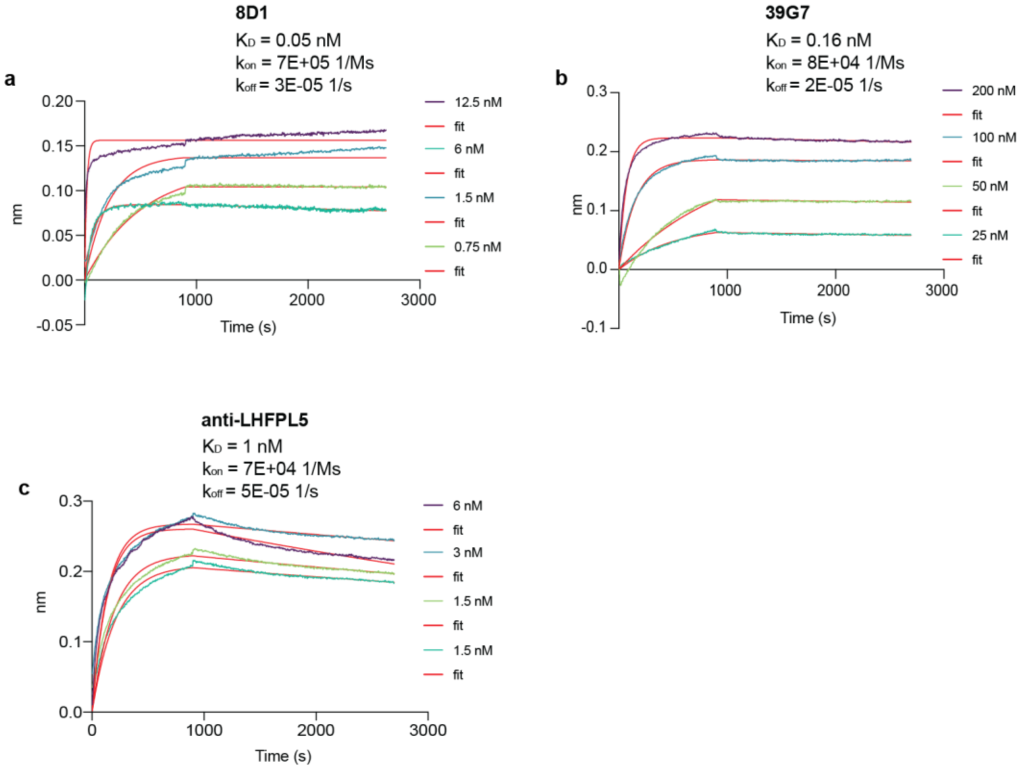


**Supplementary Figure 1: Bio-layer interferometry measurements of anti-PCDH15 and anti-LHFPL5 antibodies.**

Experimental traces are shown for anti-PCDH15 8D1 **(a),** anti-PCDH15 39G7 **(b),** and anti-LHFPL5 **(c).** Concentrations of monoclonal antibody ranged from 0.75 – 200 nM depending on the antibody.

bioRxiv preprint doi: https://doi.org/10.1101/2023.12.11.571162; this version posted December 12, 2023. The copyright holder for this preprint (which was not certified by peer review) is the author/funder, who has granted bioRxiv a license to display the preprint in perpetuity. It is made available under aCC-BY 4.0 International license.

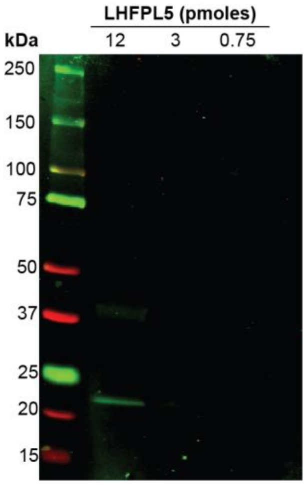


**Supplementary Figure 2: Western blot of recombinant LHFPL5.**

Recombinant LHFPL5 was probed with anti-LHFPL5 monoclonal antibody at 0.75 pM, 3 pM, and 12 pM, to facilitate direct comparison to SiMPull and SiMoA experiments.
